# Switch from 200 to 350 CD4 baseline count: what it means to HIV care and treatment programs in Kenya

**Published:** 2012-07-23

**Authors:** Joseph Mwangi, Zipporah Nganga, Raphael Lihana, Nancy Lagat, Joyceline Kinyua, Joseph Muriuki, Alex Maiyo, Florence Kinyua, Fredrick Okoth, Solomon Mpoke

**Affiliations:** 1 Centre for Virus Research (CVR), Kenya Medical research Institute (KEMRI), Nairobi Kenya; 2 Institute of Tropical Medicine and Infectious Diseases (ITROMID), Jomo Kenyatta University of Agriculture and Technology (JKUAT); 3 Department of Viral Infections and International Health, Graduate school OF Medical Sciences, Kanazawa University, Japan; 4 Kenya Medical Research Institute, Nairobi Kenya (KEMRI)

**Keywords:** CD4, New criteria, HIV, AIDS, care and treatment, ARV initiation

## Abstract

**Introduction:**

With the increasing population of infected individuals in Africa and constrained resources for care and treatment, antiretroviral management continues to be an important public health challenge. Since the announcement of World Health Organization recommendation and guidelines for initiation of antiretroviral Treatment at CD4 count below 350, many developing countries are adopting this strategy in their country specific guidelines to care and treatment of HIV and AIDS. Despite the benefits to these recommendations, what does this switch from 200 to 350 CD4 count mean in antiretroviral treatment demand?

**Methods:**

A Multi-centre study involving 1376 patients in health care settings in Kenya. CD4 count was carried out by flow cytometry among the HIV infected individuals in Kenya and results analyzed in view of the In-country and the new CD4 recommendation for initiation of antiretroviral treatment.

**Results:**

Across sites, 32% of the individual required antiretroviral at <200 CD4 Baseline, 40% at <250 baseline count and 58% based on the new criteria of <350 CD4 Count. There were more female (68%) than Male (32%).Different from <200 and <250 CD4 baseline criteria, over 50% of all age groups required antiretroviral at 350 CD4 baseline. Age groups between 41-62 led in demand for ART.

**Conclusion:**

With the new guidelines, demand for ARVs has more than doubled with variations noted within regions and age groups. As A result, HIV Care and Treatment Programs should prepare for this expansion for the benefits to be realized.

## Introduction

The new World Health Organization (WHO) guidelines 2010, on initiation of Antiretroviral Treatment (ART) recommend a switch from [1, 2]. As a result, developing countries are currently mainstreaming this recommendation in their care and treatment programs. This recommendation comes with benefit especially in Africa, where most of Human Immunodeficiency Virus (HIV) infections worldwide occur [3]. With more than twenty million HIV-infected individuals, two-thirds of all new Infections, and three-quarters of all HIV-related deaths being in Africa [4], HIV care and treatment programs continue to experience major challenges especially in universal access, early detection of infections expansion of care services ,referrals and retention of clients on care services.

With the rapid scale up of prevention programs in Africa over the last five years, many more individuals are getting tested for HIV and therefore an increase in number of identified cases. There were more than 2 million people on Highly Active Antiretroviral Therapy (HAART) in sub-Saharan by 2007 [5] and the number of individuals requiring treatment among those tested positive for HIV today is much more since access to testing, care and treatment has continued to expand.

With the implementation of new approaches driven by interventions like male circumcision [6, 7] ,the United Nations General assembly(UNGAS) new goals for zero new HIV infections, zero discrimination and zero Acquired Immunodeficiency Syndrome(AIDS)-related deaths for 2015 [8], new guidelines on treatment initiation [1], possible implementation of self testing, prevention by treatment [10, 11] and tuberculosis and HIV integration among other interventions, currently suggested or underway, it is likely that more individuals will be put on care and treatment if resource are directed in this direction.

With a prevalence of 7.4% [11], Kenya is among countries with high HIV and AIDS burden. As of 2007, there were 1.4 million Kenyans, living with HIV and an estimated 190,000 of them receive ART, representing 44% of those in need of treatment [12]. Like in many other developing countries, immunological monitoring by CD4 counts remains the main determinant for initiation and monitoring of ART in Kenya. While the number of individuals requiring but not accessing Anitretrovirals (ARVs) remained high in Kenya [12], there is limited data demonstrating the increase in demand for ARVs in the wake of the new CD4 count guidelines.

In this study, we evaluated CD4 counts distribution among HIV patients in different regions of the country in order to determine the impact on treatment demand arising from the change in criteria from

## Methods

### Study Population

This study utilized laboratory data obtained during CD4 testing from patients attending health care services in 9 selected public health facilities across the country, within a four month period- January to May 2011.These were adult patients attending comprehensive care services upon referral from clinics and various testing points and required CD4 counting as part of their evaluation. Study sites were conveniently selected, however, efforts were made to ensure representations of regions in the country. Six out the eight regions in Kenya were represented and a total number of 1376 patients were involved in the study. Demographic data on Patients was also collected during the study period.

Data obtained was used to compare treatment demand based on the CD4 baseline categories among the HIV infected individuals across 9 study sites. Comparison of number of individuals eligible for treatment per WHO 2006 guidelines, Kenyan guidelines and the new WHO 2010 guidelines was carried out to derive differences in ART needs estimates in Kenya. In addition, collected data was also used to compare treatment demand within age groups in the study population.

### HIV Serology

The HIV sero-status and confirmation of results was determined by rapid testing according to in-country and WHO HIV testing algorithm where three rapid tests; Determine HIV 1-1/2 (ABBOT Laboratories,illnois USA ), SD Bioline HIV1/2 3.0 (Standard Diagnostics, Korea) and UniGold (Trinity Biotech, Ireland) were used. All patients with positive sero-status are normally linked to care and treatment services within the health facilities in Kenya.

### CD4 Count

Flow cytometry using FACSCalibur (Becton, Dickinson San Jose, CA USA) was used for the CD4 count according to manufacturer's instructions. Briefly; 20 µl of tri-TEST CD3/CD8/CD45 reagent was pipetted into corresponding labeled tubes. Specimens were well mixed before adding 50 µl of whole blood to the corresponding tubes. This was vortex briefly to mix and tubes incubated in the dark for 15 minutes. In the next step, 450 ul of FACS lysing solution was added, vortex and incubated for 10 minutes. This was followed by acquisition and analysis. Results were reported in cell/ul of blood.

### Statistical analysis

Data obtained from this study was analyzed using SPSS version 16.0 (statistical package for social sciences). Statistical analysis was carried out using chi-squared test. Differences between CD4 distributions with age groups, gender and sites are shown.

### Ethical considerations

This study was approved through institutional scientific and ethical research committee of Kenya Medical Research Institute. Confidentiality was maintained and results were not linked to names of clients.

## Results

### Demographic characteristics of the study population

**Figure 1:**
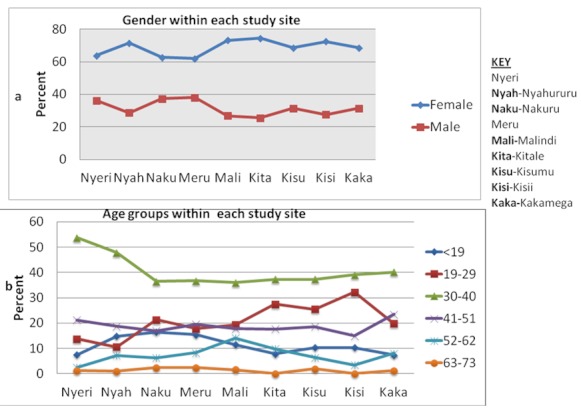
Demographic characteristic of the study population in selected health facilities in Kenya showing study population by gender, gender by sites and age groups within sites

Majority of the patients in this study (n = 1376) were female at 68% (935), with some sites (Nyahururu, Malindi, Kitale and Kisii) having over 70% of their patients being women (total; 150(71%), 142(73%), 38(74%) & 63(72% respectively) as shown in Figure 1a. In all the 9 study sites age category 30-40 had the highest number of patients at 40 % (543), range 35% -54%, with two sites; Nyeri and Nyahururu having 54 %( 43) and 48%(100) individuals within site in this category respectively (Figure 1b). Three sites, Kitale, Kisumu and Kisii had over 25% (27% , 25% & 32% respectively) of individuals in age category 19-29 years within site(Figure 1b).Age categories 52-62 years and 63-73 years had the least number of patients at less than 10% (total 126 all sites) except Malindi (14% within site).

### CD4 counts

**Figure 2:**
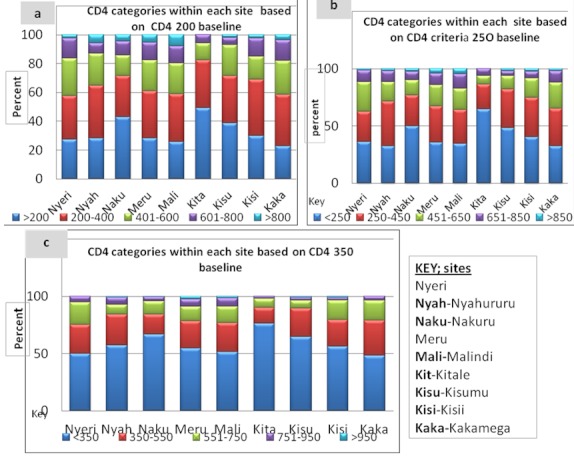
Comparisons of CD4 results from the 9 study sites in Kenya based on each baseline category; CD4 200, CD4 250 and CD4 350

Determination of CD4 count indicated that about 32% (434) of the individuals in the study population had CD4 count below 200 cell/ul (Figure 2a). Six sites out of the nine had less than 30% of their patients with counts below 200 CD4 (CD4 200 criteria) while, the remaining three sites; Nakuru Kitale and Kisumu had the highest numbers (over 30%) within site. The three sites had 43 %( 88), 49 %( 25) and 39 %( 79) of their patients with counts below 200 CD4 count baseline respectively (Figure 2a).

At criteria 250 CD4 count baseline, a total of 550 (about 40%) individuals had counts below 250 CD4 cell/ul. Comparisons at this baseline criterion indicates that all sites had over 30% of their patients with CD4 count below baseline (range 32% lowest to 64% highest) (Figure 2b). Three sites; Nakuru with 50%, Kitale 64.7% and Kisumu 48.5% had the highest individuals under CD4 250 cell/ul within site. In average, a 10% increase (from CD4 200 to CD4 250 baseline) was noted in all sites, range 7%-10% increase in 6 sites except two; Kitale (16 %- highest increase) and Nyahururu (4%- lowest increase).

At criteria 350 CD4 baseline, 58% (788) of patients had CD4 count below baseline. All the sites had above 50% of their patients at below 350 CD4 cells/ul except Kakamega at 48%. Three of the sites; Kitale at 76%, Kisumu 65% and Nakuru 66% had the highest numbers (below baseline) in this criterion (Figure 2b). There was an increase by about 15% in all sites (range 11%-18%).

When compared between the three baseline criteria, a jump from CD4 200 to CD4 350 increases those below current recommended baseline by about 26% in all sites (range 22%-29%), an increase by 354 individuals (from 434 at 200 CD4 to 788 at 350 CD4 criterion).

Analysis of the three CD4 criteria by age groups indicates that individuals below 19 years (

At CD4 250 criterion, the age group with highest number requiring treatment was 41-51 years (47%; 120 of 256 patients) followed by 52-62 years (44%; 46 of 105) and

**Figure 3:**
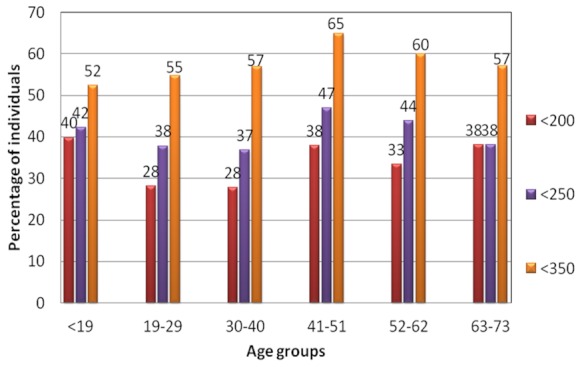
Percentage of individuals with CD4 count within age groups at CD4 count baseline 200, 250 and 350 respectively

At CD4 350 criterion, age group 41-51 had the highest demand (65%, 166 of 256 patients) followed by 52-62 (60%; 63 of 105). The lowest demand for ARVs was at age groups 19-29 years at 55% (147 of 269 patients) and below 19 years at 52 %( 87 of 166) (Figure 3c). Among the total number of patients below 350 CD4 count, age group 30-40 years had the highest number of individual requiring ART at 39% (309 patients out of 788).

Comparison on age group across the 3 baseline categories therefore indicates age group below 19 years had the highest number of individuals (40% within the age group) below CD4 200 while age group 41-51 years had the highest number of individuals below CD4 250 and 350 CD4 counts/ul (47% and 65% respectively within the each age group) (Figure 3). All age groups had over 50% of the population requiring ARVs at CD4 350 (Figure 3).

## Discussion

This study reports on CD4 distribution across regions in Kenya, among the HIV infected in line with the latest WHO HIV treatment guidelines. To our knowledge, it is also the only study of its kind showing distribution of CD4 Across age groups and regions in Kenya. Providing baseline data on the geographical distribution of CD4 across different regions in Kenya and identification of patterns in age categories is a strong merit in this study. In addition, the aim of the study in the context of scaling up of ART globally beyond the WHO 2010 criteria and to approach treatment as prevention under limited resources and fragile health systems is timely and appealing.

The WHO guidelines recommend among other specific guides, a switch from [13] to <350 CD4 count for initiation of ART [1]. While a number of laboratory tests, including antibody testing, CD4 cell count, viral load, Complete blood count, chemistry profiles, serology for hepatitis co-infections and genotypic resistance testing are required for the management of HIV and AIDS, these tests are not universally available or accessible in resource restricted settings. As a result, CD4 count and a few available tests are used in the standard of care for HIV and AIDS mostly due to cost implications. In resource restricted settings, therefore, CD4 count may remain one of the most important criteria for decision to initiate Antiretroviral for long.

In Kenya, CD4 count is among the most available test for HIV management. This is mostly due to networking of sites performing CD4 with those without CD4 testing facilities. This test, therefore, together with clinical staging has for long remained the backbone of decisions to initiation and monitoring of ART. The country, had for long adopted a CD4 250 criterion instead of 200 CD4 for initiation of ARVs [14].

As shown in this study, most of the patients were female (68 %, with some sites recording up to 74% female) compared to male (32%) (Figure 1), this variation could be due to a higher HIV prevalence among females. Differences in HIV prevalence between gender has been shown in Kenya previously, where prevalence is higher among female (8.8%) compared to male (5.5%) [12]. Maternal and child health clinics also provide a catchment for women especially through prevention of mother to child transmission (PMTCT) HIV program. As a result, probably more females are accessing care and treatment compared to males. This phenomenon has also been reported elsewhere [15, 16].

Comparison between the CD4 guidelines indicated that 32%, 40% and 58% of the study population (n = 1376) at below CD4 200, 250 and 350 cut offs, respectfully, required ARVs at the time of the study. These results demonstrate about 10% increase in the number of individuals qualifying for initiation of ARVs across the sites with each shift from 200 CD4 to 250 CD4, 15% increase from 250 CD4 to 350 CD4 cut off and about 26% increase with shift from 200 CD4 350 respectively. The criterion in Kenya has been to initiate ART at 250 CD4 counts, which was ahead of the WHO, previous recommended 200 CD4 count. With these criteria, about 250,000 patients were on ART in Kenya by December 2008 and consequently increased to about 340,000 by December 2009, a 34% increase [17]. About 520,000 HIV patients were in need of ARV Treatment in Kenya by 2009 based on WHO 2006 guidelines. Under the current WHO guidelines, the 2009 coverage translates to 48%, meaning currently over 700,000 Kenyans are in need of ARVs.

Based on the findings of this study, about 26% increase on those requiring ARV is expected with the new criteria of 350 CD4 baseline. Thus over 650,000 patients in Kenya qualify for ARVs to date which is just slightly short of the over seven hundred thousand, WHO estimates. Under the 65% ARV coverage of 2009, Kenya fell short of 80% coverage required to achieve universal access to antiretroviral therapy. With the new criteria therefore, the country falls further downwards to 48% coverage, creating a sizeable gap in meeting demand for ARVs.

As seen from the results, there are significant variations in the number of individuals qualifying for initiation of ARVs within sites at CD4< 200 and CD4 250 (Figure 2, p 0.001) and CD4< 350 (Figure 2, p 0.004) and within age groups (Figure 3). There is however no significant difference between sexes across the three CD4 baseline categories, hence a proportionate number of males should also be accessing care and treatment. At CD4 200 Kitale, Nakuru and Kisumu had the highest number of individual requiring ARVs (with 49%, 43% and 39% respectively).This scenario is replicated at CD4 250 (with 64%, 50% & 49%) and at CD4 350 classifications (76%, 67, 65%) respectively.

The prevalence of HIV by regions in Kenya indicate Nyanza(where Kisumu is located),Nairobi, Coast and Rift Valley were leading by 15%,9%, 8% and 7% [12]. Possibly the increased demand for ARVs reflects the higher HIV prevalence in these areas. The two regions (Nyanza and Rift valley) represented by the three sites have been shown to contribute to almost 50% of new HIV infections in Kenya [12]. However, Nakuru and Kitale are cosmopolitan and highly populated areas with robust ART management programs. These two areas are also highly agricultural productive. It would be expected that treatment for opportunistic infections, reduction in viral load and dietary requirements are mitigated. Increased number of individuals requiring ARVs and variations within regions therefore remains a subject of further research. Probably a population based baseline CD4 count, tracking and follow-up for ARVs enrollment and adherence to care and treatment and frequency and types of opportunistic infections in relation to CD4 count are important research questions to be addressed in understanding the variations in demand for ARVs within CD4 criteria across the regions in Kenya.

An interesting pattern was noted in the distribution of CD4 in the three categories within age groups where at CD4 200 category, age group below 19 years had the highest percentage of individual requiring ARVs while age groups 19-29 and 30-40 had the lowest at both CD 200 and 250 categories. This however changes at CD4 250 baseline where age group 41-51 years, 52-62 years and <19 years led in ARVs demand in that order. The two age groups (41-51 and 52-62) also led in demand at CD4 350 category. Probably at below 19 years individuals have their CD4 being depleted more rapidly while lower demand for ARVs at subsequent two age groups could be as a result of new infections occurring mostly in this age groups, hence the individuals still have their CD4 counts intact. The high demand for ARVs at age group 41-62 years could be as a result of long infections acquired earlier. While variations in CD4 count between sexes [18, 19],in case of infections [20],inter-laboratory [21] and between HIV positive and negative individuals [22] have been reported, in addition we report on variation within age categories as seen in this study.

In Kenya, the pattern of HIV prevalence is lowest at ages 15-19 years (below 4%) and increases gradually to about 8% and 10% at ages 20-24 and 25-29 respectively. The prevalence peaks at age group 30-34(about 14%) and age 35-39(12%) then reduces drastically with age groups above 40 yrs [12, 23]. Thus the lower demand for ARV at ages 19-40 as seen from this study, could be as a result of recently acquired infections, hence the individual's immune system is relatively intact.

As noted from this study, all age groups had over 50% of the population requiring ARVs at CD4 350 classification (Figure 3).The consequence of this increased demand for care and treatment will be overstretched health care systems in developing countries, facilities will be filled beyond capacity and task shifting due to shortage of health care workers, which is already glossily affected by rapid expansion of testing services will have to take a new dimension. Probably one of the greatest challenges in translating the new recommendations to gains against the epidemic will be initiation and retention of significant population of deserving individuals on antiretroviral. While efforts towards addressing treatment challenges are suggested [24, 17], more aggressive approaches will be required to fast track these recommendation especially in sub-Saharan Africa.

Despite the challenges ahead, treatment intervention therefore would significantly reduce pockets of particular age groups driving the epidemic as could be the case with missed opportunities like when treatment intervention is at below 200 CD4 guidelines. Implementation of the WHO guideline will therefore ensure majority of individuals across all age groups are put on ARVs. This will have impact in controlling morbidity and mortality [25, 27, 27] and at the same time ensure reduced transmission and consequently a reduction of new infections. Treatment to prevent transmission would be another gain in administering ARVs to this demanding population as shown in the CD4 350 criterion in this study. Prevention by treatment has been suggested by a number of studies [28, 29, 10, 11]. The key pointer to this is reduction of viral load which is an important factor in HIV transmission.

### Unanswered questions and future research

This study demonstrates variations in CD4 distributions across sites in Kenya, even where the regions have similar geographic and climatic conditions. Differences were also noted in the number of females’ attendance to HIV care services compared to males as well as variations in demand for ARVs by CD4 counts across age groups. Further research may be required to explore these variations.

## Conclusion

We have shown in this study that the demand for ARVs has more than doubled and consequently, the comprehensive care programs at the sites and across Kenya should prepare for the increased number of individuals due to seek HIV and AIDS care services. While this huge demand for ARVS will definitely have a huge economic and service constrains, the cost effective benefits will be realized in the long run. Probably one major approach to overcome the handles ahead is to focus on health systems strengthening in order to build sustainable comprehensive health care system to cope with the increasing demand for care. The HIV programs in the country, donors, development partners and stakeholders have an even bigger task of bridging the gap towards universal access. One major problem with HIV and AIDS services has been alienations from other healthcare services. While this problem is being addressed currently through integration of services, it will take time before systems adopt and significant gains are realized. Fragmented health care delivery systems are a hindrance towards realization of care and treatment goals. Testing for HIV, prevention and care is one continuum and any programming effort in this direction would add to overall sustainability of the health care in developing countries.
